# CAPABLE ADAPTATIONS: MEETING THE NEEDS OF OLDER ADULTS ACROSS DIVERSE CONTEXTS TO SUPPORT FUNCTION

**DOI:** 10.1093/geroni/igae098.0584

**Published:** 2024-12-31

**Authors:** Thomas Cudjoe, Lesley Steinman

**Affiliations:** Chair; Discussant

## Abstract

CAPABLE is a time-limited, person-directed, home-based program that addresses and seeks to improve the function of individuals living with disability. This effective program integrates the services of an occupational therapist, a registered nurse, and a handy worker who, in collaboration with an older adult, sets goals and creates action plans. In multiple trials nationwide, it has improved daily function and reduced disability for participants while saving health care costs. It is now being implemented in 23 States at 45 sites nationwide. Building upon the success of the original CAPABLE program, our symposium will present descriptions and findings from innovative adaptions of CAPABLE to 1) improve the health, independence, and safety of care partners, 2) promote social connection in individuals who have constrained life space or are homebound, 3) address the needs of older adults awaiting kidney transplants, and 4) improve the quality of life for older adults experiencing cognitive impairments. Presenters will provide insight into lessons learned from each CAPABLE adaptation, including perspectives on leveraging opportunities and utilizing synergy to address socio-behavioral and functional needs across these diverse contexts and populations.

